# Are physicians aware of their role in tobacco control? A conference-based survey in Portugal

**DOI:** 10.1186/1471-2458-14-979

**Published:** 2014-09-20

**Authors:** Sofia B Ravara, Miguel Castelo-Branco, Pedro Aguiar, Jose M Calheiros

**Affiliations:** Health Sciences Research Center, Faculty of Health Sciences, University of Beira Interior, Av. Infante D. Henrique, 6200-506 Covilha, Portugal; CHCB University Hospital, Quinta do Alvito, 6200-251 Covilha, Portugal; National School of Public Health, New University of Lisbon, Avenida Padre Cruz, 1600-560 Lisbon, Portugal; National Institute of Health Doutor Ricardo Jorge, Avenida Padre Cruz, 1649-016 Lisbon, Portugal

**Keywords:** Tobacco control, Smoking, Physicians, Doctors, Smoke-free policies, SHS, ETS, Training

## Abstract

**Background:**

The crucial role of physicians in tobacco control (TC) is widely recognized. In 2008, Portugal implemented a non-comprehensive smoke-free policy (SFP). In 2009, a conference-survey was carried out to explore Portuguese physicians’ engagement in tobacco control, by evaluating the following: 1) attendance at TC training and awareness of training needs; 2) participation in TC activities; 3) attitudes and beliefs regarding SFPs.

**Methods:**

Questionnaire-based cross-sectional study conducted during two major national medical conferences targeting GPs, hospitalists, and students/recent graduates. Descriptive analysis and logistic regression were performed.

**Results:**

Response rate was 63.7% (605/950). Of the 605 participants, 58.3% were GPs, 32.4% hospitalists, 9.3% others; 62.6% were female; mean age was 39.0 ± 12.9 years. Smoking prevalence was 29.2% (95% CI: 23.3-35.1) in males; 15.8% (95% CI: 12.1-19.5) in females, p < 0.001. While the overwhelming majority of physicians strongly agreed that second-hand smoke (SHS) endangers health, awareness of SFP benefits and TC law was limited, p < 0.001. A significant minority (35.5%) believed that SHS can be eliminated by ventilation systems. Most physicians lacked training; only a minority (9.0%) participated regularly in TC. Training was the most consistent predictor of participation in TC. General agreement with SFP was high; but significantly lower for indoor leisure settings, outdoors bans in healthcare/schools settings and smoking restrictions in the home/car, p < 0.001. Smoking behaviour strongly predicted support for smoking restrictions in restaurants and bars/discos, healthcare outdoors and private settings.

**Conclusions:**

The findings suggest that Portuguese physicians are not aware of their role in tobacco control. Poor engagement of physicians in TC may contribute to the current lack of comprehensive policies in Portugal and Europe and undermine social norm change. Medical and professional continuing education on tobacco control should be made top priorities.

## Background

The tobacco epidemic continues to expand globally and remains a leading cause of morbidity and premature death [1]. The World Health Organization (WHO) estimates that tobacco use and exposure to second-hand smoke (SHS) will cause over 8 million deaths in 2030 unless urgent action is taken [2]. Tobacco remains, however, the most readily preventable cause of death. In order to curb the pandemic, WHO recommends the implementation of comprehensive tobacco control (TC) policies, including price policies, and smoke-free policies (SFPs); bans on tobacco advertising, promotion and sponsorship; health education on the hazards of tobacco use and access to tobacco dependence treatment; regulation of tobacco products and other evidence-based interventions. These are based on the WHO Framework Convention on Tobacco Control (WHO-FCTC) [2]. Currently, all EU countries have ratified the WHO-FCTC. Despite this commitment, most European countries have failed to implement comprehensive policies due to tobacco industry interference in policy-making and inconsistent advocacy; underfunding and poor enforcement of TC policies [3–6].

In fact, TC progress remains deadly slow in several European countries such as Portugal [3, 6]. In January 2008, Portugal enacted a TC law [7]. A non-comprehensive and poorly-enforced smoke-free policy (SFP) was implemented disregarding FCTC guidelines. Many exemptions apply, particularly in hospitality venues, and frequent breaches have been reported [8, 9]. The role of healthcare professionals (HCPs) and its organizations has been highlighted by the WHO. Among HCPs, TC efforts should discourage tobacco use, disseminate TC training and promote smoke-free environments and other evidence-based strategies. Similarly, physicians should publicly lead TC advocacy and monitor policy impact over time [10, 11]. The roles of physicians as both exemplars and leaders are, moreover, crucial to the TC movement and to social norm change. Note too that TC progress has been greater in countries where few physicians smoke and their commitment to public health policy is strongest [10, 12]. Given this evidence, physicians’ TC awareness and training should be top priorities. Although this matter is a TC and a research relevant goal, knowledge gaps persist. This may be partially explained by the fact that surveying physicians is particularly challenging. In survey research, physicians have been appointed as a professional group from which it is difficult to obtain collaboration. Low response rates reduce sample size and can allow non-response bias and uncertainty regarding the survey findings [13, 14]. Several authors have consistently reported low response rates when surveying Portuguese physicians’ smoking behaviour and TC attitudes and practices [15–18]. The Portuguese tobacco law highlights the need to engage all HCPs, and particularly physicians, with TC activities, including training [7]. To date, little information is available concerning this requirement’s implementation. In 2009, a conference-survey was carried out to explore Portuguese physicians’ smoking behaviour trends and their engagement in tobacco control [19]. The purpose of the present study was to explore Portuguese physicians’ engagement in TC, by evaluating the following issues: 1) attendance at TC training and awareness of training needs; 2) participation in TC activities; 3) attitudes and beliefs regarding SFPs/SHS; and support for comprehensive SFPs. Additionally, the study aimed to identify factors associated with physicians’ participation in TC and their support for comprehensive SFPs.

## Methods

### Study design, study population, site and sampling, ethical approval

This was an exploratory cross-sectional study, conducted in 2009 during two major national medical conferences using a purposive-sampling procedure, and following Nardini et al’s methodology [20]. All data were self-reported. Self-administered questionnaires were delivered and collected during conferences targeting general practionners (GPs), hospital-based physicians or hospitalists, and undergraduate medical students and recent-graduates (SRGs). These conferences were the following:

1. Portuguese Stroke Society Annual Conference where questionnaires were distributed to all registered physicians (n = 450);
2. Portuguese GP Society Annual Conference where questionnaires were distributed to a systematic random sample of the attendees (33% out of 1500; n = 500).

GPs should be more engaged with tobacco control activities, according to national and international guidelines [7, 21]. In order to contrast GPs with hospital-based physicians, and to include physicians from all over the country with a wide range of age and professional development, this setting was selected. The theoretical sample size was 500 physicians assuming an expected smoking prevalence of 22.0% [22], with a 95% confidence interval (CI) and a precision of 3.6%. A total of 950 questionnaires were delivered, assuming an expected response rate of 60% [20]. The questionnaire included a cover letter explaining the study’s aims, the institutions involved, the researchers’ contact details and guarantees as to anonymity. Approval for the survey implementation was obtained from both conferences board committees. The organizing committees were previously contacted, in order to sound about the characteristics of attendees (number, specialty, and region). The study was approved by the Beira Interior University-Hospital Research Ethics Committee.

### Questionnaire and measures

Physicians’ beliefs and attitudes to SFP were the main outcomes, as those measures are strongly associated with tobacco social norms [23]. Additionally, physicians’ participation in TC was assessed. A validated questionnaire was adapted [16]. Additional items were developed addressing TC attitudes and beliefs and pilot tested among a small group of GPs, hospitalists and SRGs. The questionnaire collected standard information on socio-demographics and specialty. The second part of the questionnaire explored smoking behaviour, attitudes to being role-models (RMs) as non-smokers (RM attitudes) and smoking in private settings. The third part addressed TC practices, attitudes and beliefs, and training in smoking prevention/treatment. Smoking status was self-reported and categorized according to WHO guidelines for tobacco use [24]. Smoking in private settings was assessed by the following questions: Do you usually smoke 1) in the home 2) in the car? Answer: never (0); yes, only at an open window (1); yes, sometimes (1); yes regularly (1). Specific training in smoking prevention/cessation was assessed as undergraduate (UGT) or graduate (GT) and quantified in hours (h): (<5 h; 5–8 h; >8-12 h; >12 h). Physicians’ TC practices were assessed by the following question: Do you participate in or have you ever participated in 1) smoking prevention/TC activities 2) smoking cessation activities? Answer: regularly (1); occasionally (1); never (0). Response categories were re-coded and dichotomized into “no” (0) and “yes” (1). TC attitudes and beliefs were assessed by four items:

1. Awareness of training needs. Question: Do you consider that you need specific training in smoking prevention/treatment?
2. SHS and SFP beliefs. The wording of questions is shown in the Results section (see tables).
3. Smoking restrictions in private settings. Questions: Do you allow smoking 1) in your home 2) in your car?
4. Support for SFP in public settings. Question: Please state your agreement regarding smoking restrictions in the following settings.

Response options to item 1 and 3 were dichotomized (yes/no). Answers to items 2 and 4 were scored on a three-point and a four-point scale, depending on the contents; answer categories and re-codification is shown in data analysis and Results sections (see tables).

### Data analysis

Categorical variables are presented as absolute and relative frequencies with 95% CI, while quantitative variables are presented as mean and standard deviation (SD). Bivariable analyses were conducted using chi-square, McNemar, and Man-Whitney tests, and crude odds ratio (OR) where suitable. All associations between variables of interest were tested. Statistical analyses were conducted using SPSS-19 statistical software. A two-sided p value < 0.05 was considered to be statistically significant. Multiple logistic regression analysis (MLR) was conducted to investigate factors associated with training needs awareness (1-yes/0-no), participation in TC activities (1-yes/0-no) and strong support for SFPs (1-strongly agree/0-others). Never smokers and ex-smokers were aggregated as non-smokers. The following independent variables were tested in the bivariable analysis and included in the MLR-models: gender (male/female), age (<45/≥45 years), specialty (GPs/hospitalists), GT/UGT attendance (1-yes/0-no); RM attitudes as non-smokers (1-most positive/0-others), SHS beliefs (SHS is the major indoor pollutant: 1-strongly agree/0-others; ventilation is effective for eliminating SHS: 1-strongly disagree; disagree/0- others); smoking behaviour (smokers/non-smokers) and having a smoke-free home/car (allowing smoking in the home/car: 0-yes/1-no). A backwards stepwise procedure was set at the 0.05 significance level. Results were presented as adjusted ORs (aORs) with 95% CIs.

## Results

### Response rate and socio-demographics

Overall response rate was 63.7%: 605/950. Of the participants, 62.6% (379) were female; 58.3% (353) GPs, 32.4% (196) hospitalists, and 9.3% (56) medical students and recent graduates; mean age was 39.0 ± 12.9 years (range: 21–70). Hospitalists include 3 main specialities: internal medicine (n = 90);, neurology (n = 54); rehabilitation medicine and others (n = 52). GPs were significantly older than hospitalists (mean age ± SD: 42.1 ± 12.6 versus 37.7 ± 12.2 years; p < 0.001).

### Smoking behaviour and smoking restrictions in private settings

Smoking behaviour trends were analysed elsewhere [19]. Smoking prevalence was 29.2% (CI: 23.3-35.1) in males; and 15.8% (CI: 12.1-19.5) in females, p < 0.001. Current smoking was similar among GPs, hospitalists and SRGs [19]. Of the smokers, 52.4% (95% CI: 43.7-61.1) admitted smoking in the home and 46.8% (95% CI: 38.1-55.5) in the car (26.2% did not answer). Smoking restrictions in the home were significantly less reported than in the car: 76.5% (95% CI: 73.12-79.88) versus 84.0% (95% CI: 81.08-86.92); p < 0.001; (2.8% and 3.1% respectively did not answer).

### Training in smoking prevention/cessation

Participants’ attendance at UGT or GT is shown in Table 1. The great majority of physicians reported little or no UGT, particularly the older ones. Of those who reported UGT, 56.6% (95% CI: 49.2-63.9) have received less than 5 hours; 15.0% (95% CI: 9.7-20.3) more than 12 hours (not shown). About half of GPs reported GT, contrasting with hospitalists. Of those participants reporting GT, 40.6% (95% CI: 34.4-46.8) received less than 5 hours; 29.5% (95% CI: 23.8-35.2) more than 12 hours (not shown). Awareness of training needs was more frequently reported by GPs, non-smokers, under-45s, those reporting GT and females (Table 1). Workplace training programmes were reported more often by GPs than hospitalists (OR: 3.6; 95% CI: 2.4-5.4; p < 0.001) (not shown).

**Table 1:** **Tobacco control training attendance; awareness of training needs and associated factors**

Training	***Yes n(%)***	***95% CI***	***No n(%)***	***95% CI***	***Miss n(%)***	***Total(n)***	p value	***Crude OR***	95% CI	p value
*UGT* <45 ys	155(42.0)	37.0-47.0	212(57.5)	54.5-64.5	2(0.5)	369		*UGT*		
*UGT* ≥45 ys	18(7.6)	4.2-11.0	217(91.9)	88.4-95.4	1(0.4)	236		8.8	5.2-14.8	<0.001
*UGT* Overall	173(28.6)	25.0-32.0	429(70.9)	67.3-74.5	3(0.5)	605	<0.001	*Refer:* ≥45 ys		
*GT*-GPs	188(53.3)	48.1-58.5	164(46.5)	41.3-51.7	1(0.3)	353		*GT*		
*GT*-Hs	42(21.4)	15.7-27.1	152(77.6)	71.8-83.4	2(1.0)	196		4.2	2.8-6.2	<0.001
*GT*-overall	230(41.9)	37.8-46.0	316(57.6)	53.5-61.7	3(0.5)	549	<0.001	*Refer: Hs*		
***Training Awareness***	***Yes n(%)***	***95% CI***	***No n(%)***	***95% CI***	***Miss n(%)***	***Total(n)***	***Ass factors***	**aOR**	**95% CI**	**P value**
GPs	239(67.7)	62.8-72.6	112(31.7)	26.9-36.6	2(0.6)	353	GP	4.2	2.7-6.6	<0.001
Hs	87(44.4)	37.4-51.4	101(51.5)	44.5-58.5	8(4.1)	196	Non-smoker	2.7	1.7-4.3	<0.001
SRGs	39(69.6)	57.6-81.7	17(30.4)	18.4-42.5	0(0)	56	<45 ys	2.3	1.5-3.4	<0.001
Overall	365(60.3)	56.4-64.2	230(38.0)	34.1-41.9	10(1.7)	605	GT	2.0	1.3-3.0	0.001
							Female	1.5	1.0-2.2	0.035

Ass: associate; CI: confidence interval; GPs: General practitioners; GT/UGT: graduate/undergraduate training; Hs: Hospitalists; miss: missing values; OR: odds ratio; SRG: medical students/recent graduates; ys: years; aOR: OR adjusted for gender, age (<45/≥45 years), specialty (GP/Hs), smoking behaviour (smoker/non-smoker), GT/UGT (yes/no) and RM attitudes (most positive/others).

### Tobacco control activities

Table 2 presents physicians’ participation in TC activities. Of the responders, around 9.0% reported participating regularly in TC activities [smoking prevention: n = 53 (8.8%; CI: 6.5-11.1%); smoking cessation: n = 33 (5.5%, CI: 3.3-6.7%)]. GPs reported being involved in TC more often than hospitalists, p < 0.001. Participation in TC activities was predicted by reporting GT or more favourable RM attitudes, being a GP or under 45 (see Table 2).

**Table 2:** Prevention and cessation activities participation and associated factors (N = 602)*

Prevention/TC	Yesn; %	95% CI	Non; %	95% CI	Total	***Ass factors***	OR; 95% CI	p value	aOR;95% CI	p value	MLR model
GPs	200; 56.7	51.5-61.9	153; 43.3	38.1-48.5	353	GT	6.0; 4.2-8.7	<0.001	5.0; 3.4-7.5	<0.001	*72.4%; p* < 0.001
Hs	45; 23.2	17.3-29.1	149; 76.8	70.9-82.7	194	GP	4.3; 2.9-6.4	<0.001	2.8; 1.8-4.3	<0.001	
SRGs	19; 34.5	21.9-47.1	36; 65.5	52.9-78.1	55	RM	1.7; 1.2-2.4	0.001	1.6; 1.1-2.4	0.014	
Total	264; 43.9	39.9-47.9	388; 56.1	52.1-60.1	602						
**Cessation**	**Yes** **n; %**	**95% CI**	**No** **n; %**	**95%CI**	**Total**	***Ass factors***	**OR; 95% CI**	**p value**	**aOR;95% CI**	**p value**	**MLR model**
GPs	150; 42.5	37.3-47.7	203; 57.5	52.3-62.7	353	GT	4.3; 3.0-6.2	<0.001	4.1; 2.7-6.2	<0.001	73.1%; p < 0.001
Hs	27; 13.9	9.0-18.8	167; 86.1	81.7-91.3	194	GP	4.6; 2.9-7.3	<0.001	3.8; 2.3-6.3	<0.001	
SRG	19; 34.5	21.9-47.1	36; 65.5	52.9-78.1	55	≤45 yrs	1.3; 0.9-1.8	0.180	2.2; 1.4-3.4	<0.001	
Total	196; 32.6	28.9-36.3	406; 67.4	63.7-71.1	602	RM	1.9; 1.3-2.6	<0.001	1.8; 1.2-2.6	0.007	

Ass: associated; CI: confidence interval; GPs: General practitioners; GT/UGT: graduate/undergraduate TC training; Hs: Hospitalists; yrs: years; MLR: multiple logistic regression; OR: odds ratio; RM: attitudes to being role model as a non-smoker; SRG: medical students/recent graduates; TC: tobacco control; aOR: OR adjusted for gender, age (<45 ys/≥45 ys), specialty (GP/Hs), smoking behaviour (smoker/non-smoker), receipt of GT/UG (yes/no) and RM (most positive/others). *3 (0.5%) missing.

### Tobacco control beliefs

Table 3 depicts awareness of the TC law; perception of compliance with SFP; and SFP/SHS beliefs; contrasting smokers with non-smokers. Most physicians strongly agreed that SHS is harmful (88.8%), although only half strongly reported being the major indoor pollutant (51.2%), p < 0.001; about half totally agreed that SFPs could reduce tobacco consumption (52.5%) and disease burden (52.9%), p < 0.001; over 1/3 were fully aware of the TC law (35.9%), p < 0.001; in addition, 35.5%, p < 0.001, totally agreed that SFPs could help smokers to quit; and 33.7% believed that SHS could be eliminated by ventilation. Smokers believed more often that ventilation could eliminate SHS and were less likely to report low compliance with the ban. Reporting favourable RM attitudes or extensive GT (>5 hours) and being a non-smoker (Tables 2 and 3) or female were significantly and positively associated with some TC beliefs, but not all (not shown).

**Table 3:** **Second-hand smoke and smoke-free policy beliefs by smoking behaviour**

	Totally agree		Partially agree		Disagree						
***SFP beliefs***	***NS***	***Smokers***	***NS***	***Smokers***	***NS***	***Smokers***	p-value			OR; 95% CI	Total; miss
**Comprehensive SFP:**	n (%)	n (%)	n (%)	n (%)	n (%)	n (%)					N;n (%)
Helps smokers reduce consumption	250 (52.2)	65 (51.6)	196 (40.9)	50 (39.7)	16 (3.3)	8 (6.3)	0.318				585; 20 (3.3)
Helps smokers quit	173 (36.1)	42 (33.3)	221 (46.1)	57 (45.2)	71 (14.8)	22 (17.5)	0.712				586; 19 (3.1)
Reduces TR disease/mortality	255 (53.2)	65 (51.6)	189 (39.5)	44 (34.9)	26 (5.4)	13 (10.3)	0.116				592,13 (2.1)
I am aware of SFP law	151 (31.5)	66 (52.4)	290 (60.5)	54 (42.9)	3 (2.4)	6.3 (5.5)	<0.001				594; 11 (1.8)
Current SFP is being complied with	80 (16.7)	32 (25.4)	302 (63.0)	64 (50.8)	84 (17.5)	27 (21.4)	0.025			0.59; 0.37-0.94*	589; 16 (2.6)
	**Strongly agree**		**Agree**		**Disagree**			**Strongly dis**			
***SHS beliefs***	***Non-smokers***	***Smokers***	***Non-smokers***	***Smokers***	***Non-smokers***	***Smokers***		***Non-smokers***	***Smokers***	**OR; 95% CI; p-value**	**Total;miss**
**SHS:**	n (%)	n(%)	n (%)	n (%)	n (%)	n (%)		n (%)	n (%)		N;n (%)
Endangers health	436 (91.0)	101(80.2)	38 (7.9)	24 (19.0)	0 (0.0)	0 (0.0)		0 (0.0)	0 (0.0)	2.73; 1.57-4.75*	599; 6 (1.0)
										p < 0.001	
Is the major indoor pollutant	257 (53.7)	53 (42.1)	209 (43.6)	61 (48.4)	10 (2.1)	7 (5.6)		0 (0.0)	1 (0.8)	1.53; 1.022.28*	598; 7 (1.2)
										p = 0.037	
Ventilation is effective for eliminating SHS	21 (4.4)	10(7.9)	123 (25.7)	50 (39.7)	256 (53.4)	55 (43.7)		69 (14.4)	9 (7.1)	0.47; 0.32-0.71*	593; 12 (2.0)
										p < 0.001	

CI: Confidence intervals; NS: non-smokers; OR: crude odds ratio; SHS: second-hand smoke; SFP: smoke-free policy. TR: tobacco-related. *Reference: smokers.

### SFP support

Table 4 depicts support for SFP in public settings by smoking behaviour. Strong support for SFP were reported, respectively in healthcare premises (94.7%), schools (94.4%), public administration buildings (91.4%), and workplaces (89.9%); but significantly less support was observed in leisure settings such as restaurants (70.2%), shopping malls (64.1%), and bars and discos (55.0%), p < 0.001. In addition, strong support for outdoors bans was lower, respectively in healthcare settings (58.7%) and schools (52.6%), p < 0.001. Being a non-smoker was significantly associated with stronger support for SFP in all settings, with exception of healthcare indoors. Table 5 shows factors associated with strong support for comprehensive SFPs in public settings. Reporting a smoke-free home was the most consistent predictor of strong agreement with SFPs, followed by reporting SHS beliefs, favourable RM attitudes and being a non-smoker. Smoking behaviour was one of the main predictors of SFP support within restaurants and bars/discos, healthcare outdoors (Table 5); and smoking restrictions in the home/car (not shown).

**Table 4:** **Support for comprehensive smoke-free policies in public settings by smoking behaviour**

Suport	Strongly agree			Agree		Disagree		Strongly disagree		Total	Missings	Statistical tests
	***Non-smokers***	***Smokers***		***Non-smokers***	***Smokers***	***Non-smokers***	***Smokers***	***Non-smokers***	***Smokers***	N		
***Settings***	n; %	n; %	OR*; 95% CI p-value*	n; %	n; %	n; %	n; %	n; %	n; %		n; %	McNemar p-value
Wplaces	447; 93.3	97; 77.0	4.61; 2.59-8.21 p < 0.001	25; 5.2	23; 18.3	1; 0.2	3; 2.4	1; 0.2	1; 08	598	7;1.2	0.345
*Public adm*	450; 93.9	103; 81.7	3.36; 1.81-6.25 p < 0.001	24; 5.0	19; 15.1	1; 0.2	1; 0.8	1; 0.2	0; 0.0	599	6; 1.0	*Refer*
Health I	458; 95.6	115; 91.3	2.00; 0.91-4.37 p = 0.081	15; 3.1	5; 4.0	2; 0.4	3; 2.4	3; 0.6	2; 1.6	603	2; 0.3	0.009
Health O	312; 65.1	43; 34.1	3.45; 2.27-5.24 p < 0.001	101; 21.1	29; 23.0	60; 12.5	37; 29.4	3; 0.6	12; 9.5	597	8; 1.3	<0.001
School I	459; 95.8	112; 88.9	2.89; 1.34-6.23 p = 0.005	14; 2.9	10; 7.9	1; 0.2	2; 1.6	2; 0.4	0; 0.0	600	5; 0.8	<0.001
School O	274; 57.2	44; 34.9	2.47; 1.63-3.72 p < 0.001	108; 22.5	24; 19.0	77; 16.1	39; 31.0	12; 2.5	15; 11.9	593	12; 2.0	<0.001
Rest	371; 77.5	54; 42.9	4.54; 2.98-6.89 p < 0.001	88; 18.4	42; 33.3	14; 2.9	24; 19.0	1; 0.2	2; 1.6	596	9; 1.5	<0.001
Bars/Disc	297; 62.0	36; 28.6	4.06; 2.64-6.24 p < 0.001	140; 29.2	38; 30.2	35; 7.3	39; 31.0	2; 0.4	10; 7.9	597	8; 1.3	<0.001
Shopping malls	329; 68.7	59; 46.8	2.41; 1.61-3.61 p < 0.001	130; 27.1	48; 38.1	16; 3.3	13; 10.3	2; 0.4	3; 2.4	600	5; 0.8	<0.001

95% CI: 95% confidence intervals; Disc: Discos; I: indoors; *OR: crude odds ratio (regarding strongly agree against the others category responses, reference: smokers); O: outdoors; Public adm: public administrations buildings; Refer: Reference category for comparison (McNemar test); Rest: restaurants; Wplaces: Workplaces. Non-smokers: never-smokers + ex-smokers.

**Table 5:** **Factors associated with strong support for comprehensive smoke-free policy in public settings**

Settings	Workplaces	Public adm	Healthcare I	Healthcare O	Schools I	Schools O	Restaurants	Bars/discos	Malls
***Ass factors***	**OR; 95% CI p-value**	**OR; 95% CI p-value**	**OR; 95% CI p-value**	**OR; 95% CI p-value**	**OR; 95% CI p-value**	**OR; 95% CI p-value**	**OR; 95% CI p-value**	**OR; 95% CI p-value**	**OR; 95% CI p-value**
*Non-smoker*	2.1; 1.1-4.1 p = 0.032	-	-	2.2; 1.4-3.5 p = 0.001	-	1.7; 1.1-2.8 p = 0.031	2.6; 1.6-4.2 p < 0.001	2.6; 1.6-4.1 p < 0.001	-
*SF Home*	3.6; 1.8-6.9 p < 0.001	2.8; 1.4-5.5 p = 0.003	3.3; 1.5-7.2 p = 0.003	2.9; 1.9-4.6 p < 0.001	2.7; 1.2-6.2 p = 0.021	2.1; 1.3-3.4 p = 0.002	3.1; 1.9-4.9 p < 0.001	2.5; 1.6-3.9 p < 0.001	2.4; 1.5-3.7 p < 0.001
*RM attitudes*	6.0; 2.3-15.9 p < 0.001	2.2; 1.0-4.7 p = 0.045	-	2.3; 1.6-3.3 p < 0.001	7.3; 1.7-32.1 p = 0.008	2.0; 1.3-2.9 p = 0.001	1.8; 1.2-2.8 p = 0.006	1.7; 1.2-2.5 p = 0.005	1.9; 1.3-2.8 p = 0.001
*SHS-Pollutant*	2.4; 1.2-4.9 p = 0.015	3.8; 1.7-8.2 p = 0.001	2.5; 1.1-5.9 p = 0.035	-	5.0; 1.6-15.1 p = 0.005	1.9; 1.3-2.8 p < 0.001	1.7; 1.1-2.6 p = 0.012	1.5; 1.0-2.1 p = 0.030	1.7; 1.2-2.4 p = 0.007
*SHS Ventilation*	-	2.1; 1.1-4.1 p = 0.026	-	1.6; 1.1-2.3 p = 0.016	-	-	1.8; 1.2-2.7 p = 0.007	1.6; 1.1-2.4 p = 0.011	1.9; 1.3-2.7 p = 0.001
*G Training*	2.7; 1.3-5.7 p = 0.010	2.1; 1.0-4.5 p = 0.051	-	-	3.3; 1.2-9.3 p = 0.026	-	-	-	-
*UG Training*	-	-	5.1; 1.2;22.1 p = 0.028	-	5.0; 1.1;22.1 p = 0.034	-	-	-	-
*Female*	2.4; 1.2-4.6 p = 0.010	-	-	-	2.9; 1.2-6.7 p = 0.015	-	-	-	-
*Being a GP*						1.7;1.1-2.4 p = 0.011			

Adm: administration; ass: associated; CI: confidence interval; G:graduate; I: indoors; O: outdoors; RM: role model; SHS- second-hand smoke; SF-smoke-free; UG: undergraduate; aOR: adjusted OR for age, gender, specialty, smoking behavior, SF car, SF home, SHS believes, RM attitudes to being non-smokers, undergraduate/graduate training.

## Discussion

This exploratory study suggests that few Portuguese physicians are engaged in tobacco control. While the overwhelming majority strongly agree that SHS endangers health, strong awareness of SFP public health benefits and the current TC law is limited; a significant minority believes that SHS can be eliminated by ventilation systems. Furthermore, most physicians lacked specific TC training and only a minority participated regularly in smoking prevention or cessation activities. General agreement with SFPs was high, but significantly lower for indoor leisure settings, outdoors bans and private smoking restrictions. Moreover, participants reported high smoking rates. Among smokers, smoking in the home or car was common. GPs were more involved in TC activities, including training, than hospitalists. Younger physicians reported UGT more often and being more aware of training needs than the older ones; they also reported participating more often in cessation activities. Among physicians, 2/3 recognize that they should receive training on TC, but less than 1/3 received undergraduate training and less than half reported graduate training. Training was the most consistent predictor of participation in TC activities, followed by being a GP. As underscored by other authors [16, 17, 25–31], these findings highlight the need for engaging medical and professional continuous education, as well as medical associations, with TC; and to disseminate TC training both in medical schools, and in primary care and hospital settings. Recently, Do and Bautista have surveyed TC attitudes among a world-wide large sample of medical students. They have observed that only 25% of students have received undergraduate training on TC [30]. On the other hand, two recent national surveys have observed that around half of the Portuguese GPs and a minority (4.9%) of hospitalists have received graduate training in TC; only a minority of physicians have received extensive GT or participate in cessation programmes [17, 18]. Whereas physicians receive special training to provide effective and safe health care to populations, there is a need for specific training on smoking cessation to guarantee systematic and effective cessation counselling and support [21, 25]. Similarly, specific TC training is crucial to engage physicians and other HCPs in advocacy and policy-making [10, 11, 28, 30]. It is also worth noting that training influenced few items in SFP support and SHS/SFP beliefs, suggesting that those concerned received inadequate training on TC policy. This finding also has been reported by several authors, both nationally and internationally [16, 17, 28, 30]. In fact, following the preparation of Portugal’s TC law, HCPs’ training and engagement in smoking cessation expanded to different healthcare settings. Nevertheless, training and awareness of TC policy and capacity building are seldom included in these programmes [32]. When comparing physicians’ overall support in public settings, acceptance was significantly higher where the ban is long-standing and has fewer exemptions; and also for role-model professional workplaces, i.e. healthcare and school settings. These findings are consistent with past research that has concluded that support is stronger where bans have been implemented for a longer time and where there are fewer exemptions [33, 34]; and that additionally, workplaces rules and beliefs influence support for SFP [35]. Furthermore, cross-country research has consistently shown that smoke-free bars and pubs are significantly less well accepted than smoke-free workplaces and restaurants [36]. This same trend was observed in this study; moreover, physicians reported slightly less support for smoke-free restaurants and bars than the general population [36]. Likewise, a recent wide cross-country survey has observed that European medical students were among those reporting less favourable attitudes regarding smoking bans in restaurants and bars [30]. It should be emphasized that since there is no safe level of SHS exposure, strong SFP support should apply for all settings. These findings clearly indicate that physicians are not particularly aware of public health science or more specifically of the fact that only comprehensive SFPs protect populations against SHS. Moreover, the great majority of physicians reported low compliance with the partial ban; suggesting that they should be more aware of the need for a comprehensive policy. Factors associated with stronger support for SFPs followed the same pattern as for general population. Being a non-smoker strongly predicted support for smoking restrictions in outdoors, leisure and private settings. These settings were, in turn, those with lower physicians’ support. As reported by other studies [16, 17, 28, 37, 38], these findings clearly indicate that smoking among physicians is still a major barrier to social norm change. On the other hand, reporting a smoke-free home was the most consistent factor associated with strong SFP support. In fact, regulation of smoking in the home is a strong predictor of self-enforcement and compliance with SFPs and social norm change, even among smokers [39, 40]. Furthermore, younger physicians did not consistently report stronger support for SFPs, which may suggest that they are not trained on TC policy and that the social norm did not change after the implementation of the partial ban. In sum, all these findings suggest that physicians’ attitudes to SFP are not influenced by evidence-based public health science. This, in turn, is in line with previous studies [16, 17, 28]. Physicians’ attitudes and behaviours do, however, clearly influence their clinical and public health practice [12, 20, 26, 27]. In Portugal, the great majority of physicians are not active in leading public health policy and TC advocacy. The Portuguese Medical Association is not engaged in tobacco control; an official policy on tobacco use and advocacy is lacking [41, 42]. Meanwhile, many HCPs and physicians continue to smoke [15, 16, 19, 32]. When physicians are noticed smoking in public, this reinforces smoking visibility and social acceptance, undermining social norm change [16, 19, 29]. In addition, smoking physicians are less likely to provide cessation support or to actively advocate for tobacco control policies [30]. By contrast, when smoking physicians and HCPs are enrolled in a cessation programme, this promotes their advice on tobacco cessation [43]. Similarly, physicians’ training in TC is associated to willingness to quit [19]. Thus to promote tobacco control, especially in countries where tobacco use is prevalent among HCPs, it is crucial to implement cessation programmes targeting medical/health sciences students, physicians and other healthcare workers, both in medical/health sciences schools and healthcare settings [16, 19, 28–31, 44]. These programmes should be funded and evidence-based; they should be linked to comprehensive TC training focusing on tobacco health hazards, the role of physicians/HCPs in TC, policy and capacity building and not only tobacco cessation skills. An effective health promotion/TC national strategy should prioritise comprehensive, integrated and adequately-funded TC programmes led by TC experts and actively engaging medical/health sciences schools, medical associations and HCPs networks/societies, hospital managers and healthcare systems [10, 16, 19, 28–31, 44–47]. These programmes have a key role to play, and should contemplate the following:

- Promote and enforce SFPs, including smoke-free university/hospital campus;
- Disseminate TC training among HCPs, emphasising the key role of HCPs and physicians as non-smoking exemplars and TC advocates;
- Strengthen participation of and partnership with non-governmental organisations and civil society
- Raise public awareness regarding tobacco addiction, tobacco use and SHS health hazards, and the benefits of tobacco cessation
- Promote and adequately support smoking cessation [10, 46].

### Strengths and limitations

This study has both strengths and limitations. The main limitation includes the non-random selection of physicians who have attended two national conferences; and the low sample size when considering medical specialties sub-groups. This affects the representativeness and generalisability of the study findings. Thus sample selections bias should be stressed. Nevertheless, the target population is difficult to survey, especially in Portugal. On the other hand, it is particularly challenging to obtain a representative sample of physicians including the different specialties and healthcare settings. Nationwide physicians’ databases were not available when the study was planned. Conference surveys with high response rates and low sample selection bias are described in the literature [20]. Attendees of national medical associations’ conferences are usually representative of their members; these scientific events bring together physicians from all over the country with a wide range of age and professional development [20]. This justifies the study design.

However, whereas attendees of national medical associations’ conferences are usually representative of their members, they are not representative of the whole population of Portuguese physicians. In particular, these two conferences targeted only GPs and hospitalists, mainly neurologists, internal medicine and rehabilitation medicine doctors. Theoretically, physicians attending medical conferences would be those more motivated to or involved in research and education. However in Portugal, medical conferences are highly sponsored by the pharmaceutical industry, which in turn tends to invite clinicians more engaged with pharmaceutical prescription. We may consider that these physicians are less involved with public health. Nevertheless, WHO clearly emphasises that all healthcare providers and specially physicians should be engaged with smoking cessation and TC [10]. On the other hand, because of the study’s cross-sectional design, causality cannot be established. The study relies on self-reported responses, thus social desirability bias should be stressed. Given the response rate, non-response bias should be allowed. Furthermore, responders generally include those more interested in the subject. Thus, it should be assumed that physicians’ tobacco use is underestimated, and that physicians’ involvement in TC is probably even more limited. In spite of these limitations, this survey is one of the few that explored physicians’ involvement in TC in Portugal and obtained good response rates. When compared with national and international surveys similar trends were observed. Finally, the good response rate suggests that a well-planned conference-based survey may be an innovative and good approach to survey physicians in countries where this is particularly difficult.

## Conclusions

The findings suggest that Portuguese physicians are not aware of their role in tobacco control. Furthermore, physicians’ smoking prevalence is high. This highlights the need for engaging medical schools and medical professional organisations with tobacco control and physicians’ smoking prevention. Poor engagement of physicians in tobacco control may contribute to the current lack of comprehensive policies in Portugal and Europe, and undermine social norm change. Medical associations/organisations should acknowledge their leadership role and assume a core responsibility in promoting smoke-free environments and tobacco control best practices. To achieve this, medical and continuing education on tobacco control should be made top priorities. Training should be adequately-funded, led by TC experts and include tobacco health hazards, emphasis on the role of physicians in TC, capacity building, benefits of tobacco cessation and cessation skills.

## Abbreviations

CI: Confidence interval
FCTC: Framework Convention on Tobacco Control
GPs: General practioners
GT: Graduate training in smoking prevention/treatment
HCPs: Health care providers
OR: Odds ratio
aORs: Adjusted ORs
MLR: Multiple logistic regression
RM: Role model
SD: Standard deviation
SFP: Smoke-free policy
SHS: Second-hand smoke
SRGs: Students and recent graduates
TC: Tobacco control
UGT: Undergraduate training in smoking prevention/treatment
WHO: World Health Organisation.
